# Increased CSF levels of aromatic amino acids in hip fracture patients with delirium suggests higher monoaminergic activity

**DOI:** 10.1186/s12877-016-0324-0

**Published:** 2016-08-02

**Authors:** Leiv Otto Watne, Ane-Victoria Idland, Durk Fekkes, Johan Raeder, Frede Frihagen, Anette Hylen Ranhoff, Farrukh Abbas Chaudhry, Knut Engedal, Torgeir Bruun Wyller, Bjørnar Hassel

**Affiliations:** 1 Institute of Clinical Medicine, University of Oslo, Oslo, Norway; 2 Oslo Delirium Research Group, Department of Geriatric Medicine, Oslo University Hospital, PO BOX 4950, Nydalen, N-0424 Oslo, Norway; 3 Institute of Basic Medical Sciences, University of Oslo, Oslo, Norway; 4 Edinburgh Delirium Research Group, Geriatric Medicine, University of Edinburgh, Edinburgh, Scotland, UK; 5 Department of Clinical Chemistry, Erasmus MC, University Medical Center Rotterdam, Rotterdam, Netherlands; 6 Department of Anesthesiology, Oslo University Hospital, Oslo, Norway; 7 Department of Orthopaedic Surgery, Oslo University Hospital, Oslo, Norway; 8 Department of Medicine, Diakonhjemmet Hospital, Oslo, Norway; 9 Department of Clinical Science, University of Bergen, Bergen, Norway; 10 Norwegian National Advisory Unit on Ageing and Health, Vestfold Health Trust, Tønsberg, Norway; 11 Department of Complex Neurology and Neurohabilitation, Oslo University Hospital, N-0027 Oslo, Norway; 12 Norwegian Defense Research Establishment (FFI), Kjeller, Norway

## Abstract

**Background:**

To examine whether delirium in hip fracture patients was associated with changes in the levels of amino acids and/or monoamine metabolites in cerebrospinal fluid (CSF) and serum.

**Methods:**

In this prospective cohort study, 77 patients admitted with an acute hip fracture to Oslo University Hospital, Norway, were studied. The concentrations of amino acids in CSF and serum were determined by high performance liquid chromatography. The patients were assessed daily for delirium by the Confusion Assessment Method (pre-operatively and post-operative day 1–5 (all) or until discharge (delirious patients)). Pre-fracture dementia status was decided by an expert panel. Serum was collected pre-operatively and CSF immediately before spinal anesthesia.

**Results:**

Fifty-three (71 %) hip fracture patients developed delirium. In hip fracture patients without dementia (*n* = 39), those with delirium had significantly higher CSF levels of tryptophan (40 % higher), tyrosine (60 % higher), phenylalanine (59 % higher) and the monoamine metabolite 5-hydroxyindoleacetate (23 % higher) compared to those without delirium. The same amino acids were also higher in CSF in delirious patients with dementia (*n* = 38). The correlations between serum and CSF amino acid levels were poor.

**Conclusion:**

Higher CSF levels of monoamine precursors in hip fracture patients with delirium suggest a higher monoaminergic activity in the central nervous system during delirium in this patient group.

## Background

Delirium, an acute brain dysfunction resulting in a change in cognition and attention, is often precipitated by emergency hospitalization and surgery [1]. Delirium is especially common among elderly patients, and as many as 40–50 % of acutely hospitalized patients with hip fracture develop delirium [2, 3]. Although an increased awareness of risk factors of delirium may reduce its incidence, there is no effective pharmacological treatment once delirium has developed [1, 4]. The pathophysiology of delirium is poorly understood [5].

Aromatic amino acids are precursors for the monoamines dopamine, noradrenaline and serotonin, which have important roles in attention and cognition and have thus been of interest in delirium research [6]. The rate of monoamine synthesis in the brain is determined by the concentration of aromatic amino acids, because rate-limiting enzymes (tryptophan hydroxylase and tyrosine hydroxylase) are not saturated with substrate under physiological conditions [7]. Phenylalanine and tyrosine (dopamine and noradrenaline precursors) levels have been found to be elevated in serum in patients with delirium [8–10]. Both increased and decreased serum levels of the serotonin precursor tryptophan have been reported in patients with delirium [10, 11]. However, whether such changes in serum levels translate to the central nervous system is unclear.

Amino acid levels in CSF have to our knowledge never been assessed in patients with delirium. The purpose of this study was to examine if delirium was associated with changes in the levels of amino acids and/or monoamine metabolites in CSF.

## Methods

We included hip fracture patients that were participants in Oslo Orthogeriatric Trial (OOT), a randomized controlled trial evaluating the orthogeriatric service at Oslo University Hospital [12]. Enrollment in OOT took place from September 2009 through January 2012, and all patients with a hip fracture were eligible, irrespective of age, pre-fracture function, cognitive status, and accommodation. Patients were excluded if the fracture was caused by a high energy trauma (defined as a fall from more than one meter) or if the patient was terminally ill. Trial inclusion took place in the emergency room by the orthopedic surgeon on call. The majority of patients underwent surgery following spinal anesthesia, and CSF was sampled immediately before administration of the anesthetic agent.

### Measurements and procedures

The patients were screened for delirium once daily with the Confusion Assessment Method [13] (CAM); pre-operatively and until day 5 post-operatively (all patients) or until discharge (delirious patients). CAM scores were based on an interview with the patient, including tests of cognition, attention and alertness (digit span test, orientation and delayed recall), information from close relatives and nurses, and scrutinization of hospital records from the previous 24 h. A geriatrician (LOW) and a trained research nurse performed the assessments. If the research nurse was unsure about the delirium diagnosis, the geriatrician was consulted.

An expert panel consisting of a geriatrician (TBW) and a geriatric psychiatrist (KE) decided whether the patients fulfilled the ICD 10 criteria for dementia prior to the fracture. The expert panel used all available information, including the Informant Questionnaire on Cognitive Decline in the Elderly [14, 15] (IQCODE), case notes, and cognitive tests performed at the follow up controls in the OOT (Mini-Mental state examination (MMSE) [16], clock drawing test [17], the 10 word test from the Consortium to establish a Registry for Alzheimer’s disease battery (CERAD) [18] and The Clinical Dementia Rating scale (CDR) [19]). Cases with disagreement were discussed until a consensus was reached. More details regarding the delirium assessments and other cognitive measures have previously been published [12, 20]. Proxies were interviewed regarding pre-fracture Activities of Daily living (Barthel ADL Index) [21]. Comorbid conditions were quantified using the Charlson Comorbidity Index [22]. The Acute Physiological and Chronic Health Evaluation II (APACHE II) [23] score was calculated as a measure of physiological disturbance on admission to the hospital.

### Sample collection and handling

CSF was collected in polypropylene tubes and was centrifuged as soon as possible. The supernatant was stored in aliquots of 1000 μl at −80 °C. Serum was collected pre-operatively by venous puncture. The sample tubes stood in the vertical position for 30 min at room temperature for clotting before centrifugation. Aliquots of 200–500 μl were then stored at −80 °C in polypropylene tubes.

### Sample selection

**Fig. 1:**
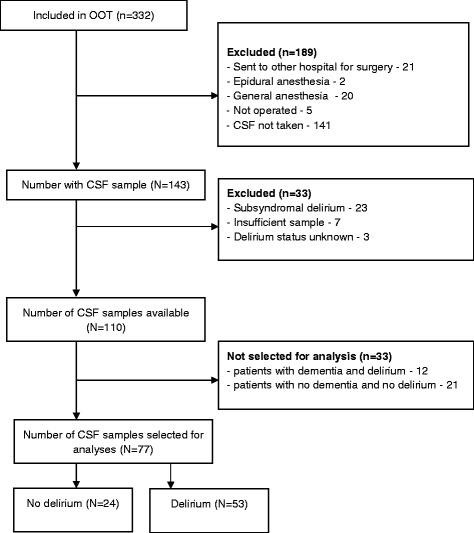
Flow diagram showing selection of CSF samples from hip fracture patients. OOT = Oslo Orthogeriatric Trial

CSF was obtained from 143 hip fracture patients (Fig. 1). We wanted to compare delirium patients with patients who were free from any delirious symptoms, and patients with subsyndromal delirium (defined as at least one positive CAM item, but never full delirium) were therefore excluded. Patients with unknown delirium status were also excluded.

We wanted all combinations of delirium and dementia status represented:

1. no delirium/no dementia
2. delirium/no dementia
3. no delirium/dementia
4. delirium/dementia

There was a surplus of samples from group 1 and 4, and we selected random samples from these two groups.

### Amino acid analysis

Samples of CSF and serum were thawed on ice, and 18 μL of CSF were mixed with 2 μL of α-aminoadipate (internal concentration standard), 200 μmol/L in sodium azide, 2 % (weight/volume), giving a final concentration of the internal standard of 20 μmol/L. Serum was mixed 1:1 with α-aminoadipate, 200 μmol/L in sodium azide, 2 %. The sodium azide was added to avoid bacterial growth. Amino acids were analyzed by high-performance liquid chromatography (HPLC) and fluorescence detection after pre-column derivatization with o-phthaldialdehyde as described by Dahlberg et al. [24]. The analyses were done blindly with respect to clinical data.

### Monoamine metabolites analysis

5-hydroxyindoleacetic (5-HIAA) and homovanillic acid (HVA) were separated by HPLC using a Zorbax Eclipse XDB-C8 column, and measured by electrochemical detection. Oxidation potential was set at 0.6 V after samples had been deproteinized with sulphosalicylic acid, as previously described [25, 26]. Quantification was done by measuring peak heights relative to the internal standard 5-methylserotonin. The 5-HIAA and HVA analyses were done blinded to clinical data. 5-HIAA and HVA were analyzed in CSF only.

### Data presentation and statistics

In our primary analyses, we compared the levels of amino acids in CSF and serum between patients with and without delirium by Mann–Whitney U tests. Since dementia is associated with neurodegeneration and the cerebral substrate for delirium might be different for subjects with or without a dementia disorder, these analyses were carried out stratified by pre-fracture dementia status. In a secondary analysis we then divided the hip fracture patients into three subgroups:

1. Ongoing delirium – patients who had delirium when CSF was taken.
2. Incident delirium – patients who were free from delirium when CSF was taken, but developed delirium later.
3. Never delirium – patients who never experienced any symptoms of delirium.

Correlations between amino acid levels in CSF and serum were evaluated with Spearman’s correlation coefficient.

To assess the relationship between the amino acids and delirium when adjusting for other covariates, we carried out logistic regression analyses. We made separate regression models for each amino acid/monoamine metabolite, however limited to those identified in univariate analyses as having the largest differences between patients with and without delirium. “Delirium anytime” was the outcome variable in all models. As covariates we included variables known to be associated with delirium; age, gender, APACHE II (Acute Physiology and Chronic Health Evaluation II), Barthel ADL (Activities of Daily Living) and CCI (Charlson Comorbidity Index) scores.

Due to the exploratory nature of this study, correction for multiple comparisons were not applied. All statistical analyses were performed using IBM SPSS Statistics version 20.

## Results

In total, 77 CSF samples were analyzed and 53 of the samples were from patients with delirium. Patients with delirium were older, had more often dementia, had higher APACHE II and more ADL impairment compared to patients without delirium (Table 1). Twenty-nine of the samples were from patients with ongoing delirium, 21 from patients with incident delirium and 24 from patients who never developed delirium (preoperative delirium status unknown in three patients).

**Table 1:** Baseline characteristics

	No delirium (*n* = 24)	Delirium (*n* = 53)	*P*-value
Age, median (IQR)	84 (70–89)	86 (81–90)	0.21
Gender, male (%)	7 (29)	16 (30)	0.93
APACHE II, median (IQR)^a^	8 (6–10)	9 (8–11)	0.10
IQCODE, median (IQR)	3.1 (3.0–3.6)	4.3 (3.3–4.8)	<0.001
Dementia (%)	3 (13)	35 (66)	<0.001
ADL, median (IQR)	19.5 (18–20)	16 (12–19)	<0.001
CCI, median (IQR)	1 (0–2)	1 (0–2)	0.96

^a^Arterial blood gas and hematocrit omitted from formula

*IQR* interquartile range, *APACHE II* Acute Physiology and Chronic Health Evaluation II, *IQCODE* Informant Questionnaire on Cognitive Decline in the Elderly, *ADL* activities of daily living, *CCI* Charlson Comorbidity Index score

### CSF levels of amino acids and monoamine metabolites

**Fig. 2:**
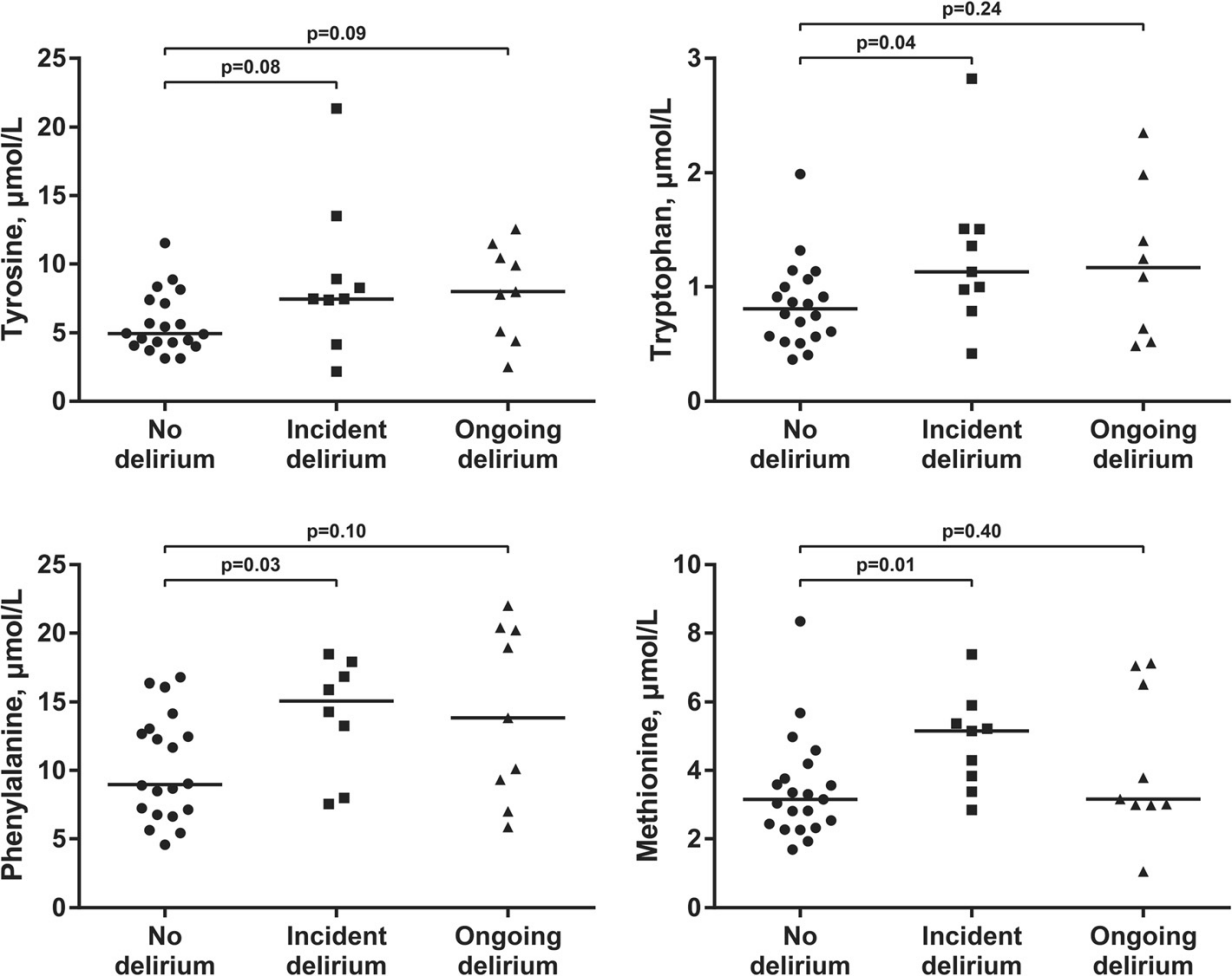
Levels of aromatic aminoacids in CSF. Levels of aromatic amino acids in CSF of hip fracture patients free from dementia, stratified on delirium status at the time of CSF sampling. The horizontal line represents the median

In patients without pre-fracture dementia (*n* = 39), those with delirium had significantly higher levels of tryptophan (40 % higher, Table 2), tyrosine (60 % higher), phenylalanine (59 % higher), and methionine (29 % higher). In patients with pre-fracture dementia (*n* = 38) the same four amino acids were also highest in delirious patients, however statistically significant only for tyrosine, phenylalanine and methionine. When the patients were stratified by delirium status at the time of sampling, we found that these amino acids tended to be highest in the group of patients with incident delirium, both in patients with and without dementia (Fig. 2).

**Table 2:** Concentration of amino acids and monoamine metabolites in CSF

	No dementia (*n* = 39)			Dementia (*n* = 38)		
	No delirium (*n* = 21)	Delirium (*n* = 18)	*p*-value	No delirium (*n* = 3)	Delirium (*n* = 35)	*p*-value
Amino acids						
Alanine, μmol/L	25 (20–36)	27 (19–43)	0.39	17 (16–17)	25 (20–32)	0.128
Arginine, μmol/L	12 (11–16)	12 (9–17)	0.53	9 (9–9)	12 (9–13)	0.25
Aspargine, μmol/L	5 (4–6)	6 (3–8)	0.51	3 (3–3)	4 (4–5)	0.07
Glutamate, μmol/L	0.7 (0.4–1.8)	0.9 (0.6–1.7)	0.44	0.6 (0.3–0.6)	0.5 (0.4–0.8)	0.91
Glutamine, μmol/L	460 (360–528)	505 (384–588)	0.34	284 (278–284)	385 (321–457)	**0.045**
Glycine, μmol/L	28 (24–35)	27 (21–38)	0.51	23 (22–23)	28 (25–36)	0.72
Isoleucine, μmol/L	5 (3–6)	6 (3–10)	0.11	5 (4–5)	6 (4–7)	0.88
Leucine, μmol/L	11 (7–15)	12 (9–22)	0.38	12 (9–12)	13 (9–16)	0.98
Lysine, μmol/L	13 (11–15)	13 (10–17)	0.77	10 (10–10)	12 (10–15)	0.48
Methionine, μmol/L	3 (2–4)	4 (3–6)	**0.030**	3 (1–3)	4 (3–5)	**0.029**
Phenylalanine, μmol/L	9 (7–13)	14 (9–19)	**0.014**	6 (6–6)	9 (7–11)	**0.021**
Serine, μmol/L	17 (14–18)	15 (10–17)	0.21	13 (13–13)	15 (12–18)	0.92
Taurine, μmol/L	8 (7–11)	8 (6–10)	0.64	8 (7–8)	7 (5–9)	0.71
Tryptophan, μmol/L	0.8 (0.6–1.0)	1.1 (0.7–1.5)	**0.042**	0.6 (0.4–0.6)	0.7 (0.6–0.9)	0.15
Tyrosine, μmol/L	5 (4–7)	8 (5–11)	**0.028**	4 (3–4)	5 (4–7)	**0.041**
Valine, μmol/L	16 (13–25)	18 (14–31)	0.32	12 (12–12)	15 (12–21)	0.47
Monoamine metabolites						
HVA, nmol/L	170 (102–280)	204 (125–250)	0.42	134 (67–134)	114 (68–179)	0.88
5HIAA, nmol/L	138 (111–156)	170 (119–261)	**0.048**	99 (81–99)	104 (68–(140)	0.84

*IQR* interquartile range, *HVA* homovanillic acid, *5HIAA* 5-hydroxyindoleacetic

Concentration of amino acids and monoamine metabolites in hip fracture patients with and without delirium, stratified by pre-fracture dementia status. All values are median (IQR). *P*-values were calculated using Mann–Whitney *U* test. P-values below 0.05 are in bold

**Fig. 3:**
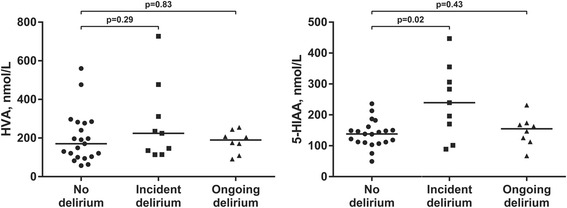
Levels of monoamine metabolites in CSF. Levels of monoamine metabolites in CSF in hip fracture patients free from dementia, stratified on delirium status at the time of CSF sampling. The horizontal line represents the median. HVA = Homovanillic Acid. 5HIAA = 5-hydroxyindoleacetic

In patients without dementia, CSF levels of 5-HIAA were significantly higher in patients with delirium, paralleling the increased level of tryptophan in this group (Table 2, Fig. 3). In contrast, the level of HVA was not significantly different between the groups. In patients with dementia there was no significant difference between patients with or without delirium, with respect to 5-HIAA or HVA.

In regression analyses, tryptophan, tyrosin, phenylalanine, methionine and 5-HIAA remained significantly associated with delirium status in patients free from dementia when adjusting for age, gender, ADL, Charlson and APACHE II (Table 3). The sample size was insufficient to carry out regression analyses in the stratum with dementia.

**Table 3:** Logistic regression controlling for potential confounding of the association between delirium and biomarkers in patients without dementia. Adjusted by age, gender, APACHE II (Acute Physiology and Chronic Health Evaluation II), Barthel ADL (Activities of Daily Living) and CCI (Charlson Comorbidity Index) scores

	Unadjusted		Adjusted	
	OR	95 % CI	OR	95 % CI
Methionine, μmol/L	1.49	0.97 to 2.27	2.26	1.12 to 4.57
Phenylalanine, μmol/L	1.20	1.03 to 1.41	1.69	1.11 to 2.58
Tryptophan, μmol/L	5.08	1.03 to 24.9	10.9	1.08 to 111.4
Tyrosine, μmol/L	1.32	1.02 to 1.71	1.94	1.09 to 3.42
5HIAA, nmol/L	1.01	1.00 to 1.03	1.01	1.00 to 1.03

*OR* odds ratio, *CI* confidence interval, *5HIAA* 5-hydroxyindoleacetic

Most amino acids were higher in patients without pre-fracture dementia than in those with dementia, and this difference was statistically significant for asparagine, glutamate, glutamine, tyrosine, alanine, tryptophan, valine, and phenylalanine. HVA (median 184 vs 118 nmol/L, *p* = 0.005) and 5-HIAA (median 147 v 104 nmol/L, *p* = 0.002) were also significantly higher in patients without chronic cognitive impairment.

When hip fracture patients with and without dementia were pooled together, the only significant difference in CSF was a higher methionine concentration in patients with delirium compared to patients without (median 4.0 vs 3.1 μmol/L, *p* = 0.002).

### Serum levels of amino acids. Correlation with CSF levels

Pre-operative serum was available for 44 patients. There were no significant differences between groups except for taurine which was higher in demented patients without delirium than in demented patients with delirium (median 8 v 7 μmol/L, *p* = 0.047). A significant correlation between serum and CSF levels of amino acids was observed only for valine (*r* = 0.38, *p* = 0.01), glutamine (*r* = 0.44, *p* = 0.003) and isoleucine (*r* = 0.37, *p* = 0.02).

## Discussion

The main finding in this study was higher CSF levels of tryptophan, tyrosine, phenylalanine, methionine and 5-HIAA in hip fracture patients with delirium and this remained a significant finding also in adjusted analyses. The highest levels of these amino acids were seen in patients with incident delirium, and this pattern was similar in patients with and without dementia. To our knowledge this is the first study of CSF levels of amino acids in patients with delirium, and the results might contribute to the understanding of delirium pathophysiology.

An increased level of tryptophan has been linked to increased formation of serotonin in the brain, because tryptophan hydroxylase, the rate-limiting enzymatic step in serotonin synthesis, is not saturated with substrate under physiological conditions (for review, see Fernstrom [27]). We also found a significantly increased level of the serotonin metabolite 5-HIAA in non-demented patients with delirium. This paralleled the increased CSF level of tryptophan in the same group, suggesting (in line with Fernstrom [27]) that an increased level of tryptophan leads to increased formation and turnover of serotonin. Our data therefore supports a notion of increased serotonergic neurotransmission in patients with delirium.

We also found that patients with delirium had higher CSF levels of tyrosine and its immediate precursor, phenylalanine. An increased concentration of tyrosine would be expected to cause increased formation of dopamine, and our findings are in line with the theory of dopamine excess in delirium [6]. Although we found an increased concentration of tyrosine, we did not see the same relationship between CSF levels of tyrosine and the dopamine metabolite HVA that we did for tryptophan and serotonin metabolite 5-HIAA. Methionine contributes a methyl moiety in the synthesis of noradrenaline from dopamine [28]. Therefore the increase in methionine could allow for an increased metabolism of dopamine into noradrenaline.

### Comparison with other studies

Our results suggest higher monoaminergic activity in delirious patients. One of the very few published animal studies in delirium gives support to our finding; in a rat model delirium was associated with increased CSF levels of the serotonine metabolite 5-HIAA and the dopamine metabolites HVA and 3,4-dihydroxyphenylacetic acid, and treatment with a selective serotonin 5HT1A antagonist reduced delirium symptoms in the same animals [29]. Further support for increased serotonergic neurotransmission in delirium comes from drug trials. A recent double-blind randomized controlled study in hip fracture patients showed that post-operative administration of the serotonin antagonist ondansetron led to a lower incidence and shorter duration of post-operative delirium compared to placebo [30]. In contrast, another randomised controlled trial found an increased risk of hyperactive delirium in patients undergoing major elective surgery who were given tryptophan postoperatively [31].

Although this is the first study of amino acids in CSF, there are several reports of changes in amino acid concentrations in serum from patients with delirium. Both higher and lower levels of tryptophan have been associated with delirium [9, 10, 32–34]. Serum phenylalanine and tyrosine have been reported to be increased in delirium [8–10]. In our study we found only minor differences between serum levels of amino acids between patients with and without delirium. The poor correlation between serum and CSF levels of amino acids in the present study illustrates the difficulty of making inferences about brain levels of amino acids from their serum levels.

CSF monoamine metabolites HVA and 5-HIAA have been analysed in one study each in delirium. A Mexican study published in 2008 included 51 patients (31 with delirium) with acute brain infection [35]. The patient cohort was different from ours (mean age 36, high prevalence of HIV). There was no significant difference in HVA levels between patients with and without delirium. Subgroup analyses revealed, however, that patients with psychotic symptoms had significantly higher levels of HVA compared to patients without such symptoms [35]. CSF levels of 5-HIAA was measured in a Finnish study of 69 patients with delirium and 14 healthy controls. 5-HIAA was higher in delirious patients than in controls, but this difference was significant only in two subgroups of patients: those with multi-infarct dementia, and those with no apparent CNS disease [36].

We found that both amino acids and monoamine metabolites were reduced in patients with dementia, as also reported in other studies [37, 38]. Since dementia is a strong risk factor for delirium, most patients with delirium had dementia and most patients without dementia were free from delirium. Since delirium seems to be associated with an increase in the same amino acids that are decreased in dementia, delirium and dementia might level each other out in analyses not taking chronic cognitive decline into account. This was clearly illustrated in our study. When patients with and without dementia were analyzed together, only methionine (in CSF) and taurine (in serum) were significantly different between patients with and without delirium. In stratified analyses, however, a similar pattern emerged in both groups.

### Strength and weaknesses

Many of the candidate biomarkers in delirium are also influenced by dementia [39] and the main strength of our study was the relatively large sample size, making it possible to select CSF samples for analyses stratified by pre-fracture cognitive status. Theoretically, it is likely that some neurotransmitters are involved in initiating delirium whereas others play a role in severity, maintaining or resolution of the delirious episode. We were able to stratify our analyses according to delirium status at the time of sampling, and thus able to demonstrate that the monoaminergic activation seems to be at the highest prior to a delirious episode. We consider this a finding of possible importance. A weakness with our study was that not all delirium assessments were done by a geriatrician. Since patients were assessed weekdays only, it is possible that brief episodes of delirium during weekends have been missed. The cross-sectional design is another weakness of our study; however, a longitudinal study with repeated CSF sampling from patients with delirium will be very hard to perform. We analyzed several amino acids, and there is an increased risk of false positive findings. Since hip fracture is an acute event, objective cognitive testing prior to the fracture is not possible. We believe our approach with a consesus based dementia diagnosis is superior to the use of IQCODE alone. Nevertheless, the retrospective classification of dementia is a weakness of our study. We also have no information regarding dementia etiology.

## Conclusion

This first study of CSF levels of amino acids in delirium suggests increased monoaminergic activity is associated with delirium in hip fracture patients.

## Abbreviations

5-HIAA, 5-hydroxyindoleacetic (5-HIAA); ADL, activities of daily living; APACHE II, the Acute Physiological and Chronic Health Evaluation II; CAM, confusion assessment method; CCI, Charlson Comorbidity Index score; CDR, the Clinical Dementia Rating scale; CERAD, Consortium to establish a Registry for Alzheimer’s disease battery; CSF, cerebrospinal fluid; HPLC, high-performance liquid chromatography; HVA, homovanillic acid; IQCODE, Questionnaire on Cognitive Decline in the Elderly; IQR, interquartile range; MHPG, metabolite 3-methoxy-4-hydroxyphenylglycol; MMSE, Mini-Mental state examination; OOT, Oslo Orthogeriatric Trial; OR, odds ratio
